# Entanglement due to Delayed Removal of a Buddy Wire

**DOI:** 10.1155/2014/513737

**Published:** 2014-12-29

**Authors:** Marc Vorpahl, Melchior Seyfarth, Klaus Tiroch

**Affiliations:** Department of Cardiology, HELIOS Klinikum Wuppertal, Witten/Herdecke University, Arrenberger Strasse 20, 42117 Wuppertal, Germany

## Abstract

A buddy wire is often used to aid in the delivery of balloons and stents when negotiating tortuous or calcified vessels. We present a planned two-stent mini-crush intervention complicated by entanglement of the buddy wire with the second stent and subsequent distortion of the stent within the guiding catheter. Based on this case, we suggest removing the buddy wire immediately after successful positioning of the first stent, because entrapment with a second stent is possible and may lead to challenging situations in a simultaneous two-stent strategy.

## 1. Introduction

Placement of a second coronary guide wire parallel to the working wire can be considered for challenging percutaneous coronary interventions (PCI). Such situations include calcified lesions, tortuous vessels, and sharp bends. This “buddy wire” stabilizes the guiding backup and position by straightening of the target vessel [1]. It is recommended to remove the “buddy wire” after stent positioning and before stent implantation to avoid entrapment of the buddy wire between the vessel and the stent. Treatment of a complex bifurcation lesion with the “mini-crush” technique using two simultaneous stents has been well described [2, 3]; however, the timing of removing a buddy wire during stent delivery is not well established.

## 2. Case Report

**Figure 1:**
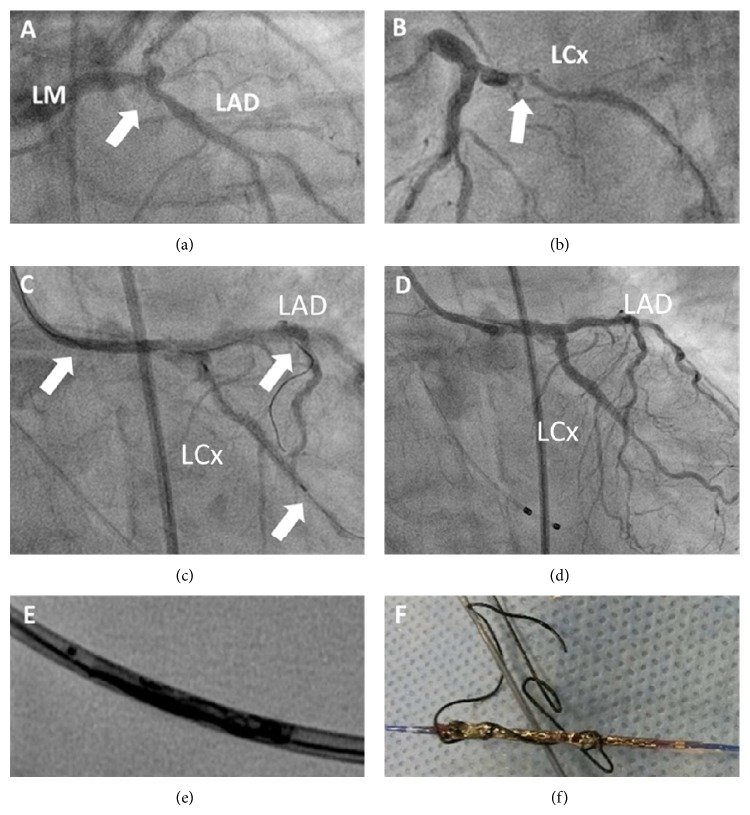
(a) and (b) Angiogram showing severe coronary artery disease with significant stenosis of the distal left main artery (LM) and the proximal left circumflex (LCx) (white arrows). (c) Mini-crush stenting was planned with one wire in the LAD and 2 wires including the buddy wire in the LCx. Advancement of the second stent into the LAD leads to entrapment and distortion of this stent by the buddy wire after successful positioning of the first stent into the LCx. (d) Provisional stenting of the LM-LCx lesion showed a good final result. (e) and (f) Attempts to pull the LCx “buddy wire” failed directly after advancement and before expansion of stents. Forced pullback of the buddy wire tore the LAD stent catheter tip off the shaft, blocking the mid-part of the 7F Guiding.

An 86-year-old female presented with acute decompensated heart failure and non-ST elevation myocardial infarction. Echocardiography demonstrated severe impaired systolic left ventricular function and moderate mitral regurgitation. Cardiac catheterization revealed severe coronary artery disease with significant stenosis of the distal left main artery (LM) and the ostial/proximal left circumflex (LCx) (Figures 1(a) and 1(b) white arrows) and intermediate stenosis of the proximal left descending artery (LAD). Based on a EuroSCORE II of 36, the Heart Team decision was in favor of a percutaneous revascularization strategy. The upfront plan was to treat the left main bifurcation with a simultaneous two-stent “mini-crush” strategy. The calcified and angulated proximal LCx required a “buddy wire” (FIELDER Coronary Guide Wire, ASAHI) in addition to the working wire (ChoICE PT Extra Support Guide Wire, Boston Scientific). Another FIELDER wire was placed into LAD. Predilation of the LAD and LCx (2.0 and 2.5 × 20 mm, NC Trek, 20 atm., Abbott Vascular) occurred smoothly, and the first stent (Xience PRO 3.0 × 28 mm, Abbott Vascular) was successfully advanced into the LCx over the ChoICE PT working wire. Then, we advanced the second stent into the LAD (Xience PRO 3.0 × 28 mm, Abbott Vascular). The advancement of the LAD stent was effortless within the guiding catheter except for the last few centimeters and within the left main, where increasing friction impeded further advancement. Surprisingly, initial attempts to pull the LCx “buddy wire” failed after successful advancement of both stents most likely related to the twisted LCx buddy wire around the LAD wire/LAD stent (Figure 1(c)). Due to entanglement, forced pullback maneuvers of the buddy wire tore the LAD stent catheter tip off the shaft, blocking the mid-part of the 7F Guiding EBU 4.0 (Medtronic) (Figure 1(e)). Fortunately, we were able to deploy left main/LCx stent without significant plaque shifting into the LAD, leaving an excellent single stent result (Figure 1(d)). The guide catheter was removed, revealing the severely entangled stent wrapped by the FIELDER “buddy wire” (Figure 1(f)).

## 3. Discussion

The “buddy wire” is a well-accepted technique for advancement of balloons and stents in calcified lesions, sharp bends, and tortuous vessels. During a complex bifurcation PCI, the first stent should be placed parallel to the buddy wire in order to gain optimal support. With a planned two-stent strategy, however, there is a certain reluctance to remove the buddy wire prematurely before final positioning of stents. In our case though, we experienced an entanglement of the buddy wire during advancement of the second stent with subsequent disruption of the stent. We therefore suggest removing the buddy wire immediately after successful positioning of the dedicated first stent, because entrapment with a second stent is possible and may lead to challenging situations in a planned simultaneous two-stent strategy.

## 4. Conclusion 

In a planned simultaneous two-stent strategy, we suggest removing the buddy wire immediately after successful positioning of the first stent to avoid entanglement of the second stent with the buddy wire.

## References

[1] Burzotta F., Trani C., Mazzari M. A. (2005). Use of a second, ‘buddy’ wire during percutaneous coronary interventions: a simple solution for some challenging situations. *Journal of Invasive Cardiology*.

[2] Colombo A., Stankovic G., Orlic D. (2003). Modified T-stenting technique with crushing for bifurcation lesions: Immediate results and 30-day outcome. *Catheterization and Cardiovascular Interventions*.

[3] Galassi A. R., Colombo A., Buchbinder M. (2007). Long-term outcomes of bifurcation lesions after implantation of drug-eluting stents with the ‘mini-crush technique’. *Catheterization and Cardiovascular Interventions*.

